# The association between television viewing time and percent body fat in adults varies as a function of physical activity and sex

**DOI:** 10.1186/s12889-019-7107-4

**Published:** 2019-06-13

**Authors:** Richard R. Suminski, Freda Patterson, Mackenzie Perkett, Katie M. Heinrich, Walker S. Carlos Poston

**Affiliations:** 1 Center for Innovative Health Research, Department of Behavioral Health and Nutrition, University of Delaware, 325 Tower at STAR, Newark, DE 19716 USA; 2 Department of Behavioral Health and Nutrition, University of Delaware, Tower at STAR, Newark, DE 19716 USA; 3 Department of Behavioral Health and Nutrition, University of Delaware, Bob Carpenter Sports Building, 26 N College Avenue, Newark, DE 19716 USA; 4 Kansas State University, Department of Kinesiology, Functional Intensity Training Lab, Natatorium 1A, Manhattan, KS 66506 USA; 5 National Development and Research Institutes, Institutes for Biobehavioral Health Research, 1920 143rd Street, Suite 120, Leawood, KS 66224 USA

**Keywords:** Obesity, Sedentary behavior, Survey research, Cross-sectional

## Abstract

**Background:**

Data suggest that sedentary behavior is an independent risk factor for obesity; however, the extent to which physical activity (PA) and sex alter this relationship remains unclear. To address this gap, the current study examined the association between television (TV) viewing time and percent body fat (%BF) as a function of PA level and sex.

**Methods:**

Trained interviewers assessed 454 adults at their place of residence. Participants completed questionnaires to determine h of TV watched per week, PA level (inactive = not meeting PA guidelines vs. active = meeting PA guideline), and covariates including demographics (e.g., sex), depression symptoms, perceived stress, fruit and vegetable intake, and environmental support for PA. Foot-to-foot bioelectrical impedance (Tanita TBF-300, Tokyo, Japan) was used to assess %BF. Mixed models were generated to examine the association between TV h/wk. and %BF as a function of PA level and sex while accounting for the multi-level nature of the data (neighborhood- and individual-levels) and covariates.

**Results:**

Participants were 44.4 ± 14.0 (Mean + Standard Deviation) years of age with 33.2 ± 11.1%BF, and watched 19.3 ± 15.5 h/wk. of TV. Most were female (70.9%) and inactive (63.2%). Mixed model regression demonstrated that among inactive participants, each additional h of TV viewed/wk. was associated with a 1.03% increase in %BF; TV h/wk. and %BF were not associated in active adults. When models were further stratified by sex, h of TV viewed/wk. were significantly associated with %BF only in inactive females. Each additional h of TV viewed/wk. was associated with an increase in %BF of 1.14%. Conclusion: Interventions targeting PA and/or TV viewing time may be a high-priority to curb excess BF accumulation especially among inactive females.

## Background

Obesity is a leading risk factor for cardiometabolic disease and cause of death world-wide [1]. The prevalence of obesity varies by race/ethnicity and age with significantly higher rates seen among non-Hispanic Black (46.8%) and Hispanic (47.0%) versus non-Hispanic white adults (37.9%), as well as in men and women aged 40–59 (40.8 and 44.7%, respectively) versus 20–39 years (34.8 and 36.5%, respectively) [2]. Demographic transitions, such as an increasing proportion of the population being older, suggests that the population burden of obesity is only poised to grow. Behavioral approaches to reduce obesity, including increased time in moderate-vigorous physical activity (PA) and improved dietary intake, have met with limited long-term success [3, 4]. To achieve public health goals of a reduction in obesity prevalence, new approaches are needed [5].

Sedentary behavior is defined as any waking behavior characterized by an energy expenditure ≤1.5 metabolic equivalents, while in a sitting, reclining or lying posture [6]. Growing evidence consistently suggests that daily sedentary time is a strong determinant of health outcomes [7]. For example, in a 15-year prospective cohort study conducted in Sweden, data from 851 adults showed that as compared to the least sedentary adults, the most sedentary had a more than five-fold increased risk of death from cardiovascular diseases [8]. Importantly, several demographic and psychological factors have been associated with increased sedentary time including male gender, not having a college degree, greater stress and more depressive symptoms [9–11]. As such, decreasing sedentary time is an important behavioral target for reducing disease risk in these population sub-groups as well as the general population [9].

One of the open questions in the study of sedentary behavior is the influence of PA on the sedentary behavior-obesity relationship. A large body of literature documents the positive association between sedentary behaviors – TV viewing in particular – and the odds of overweight and obesity, independent PA [12–16]. Some studies have shown an interactive association between PA and sedentary behavior whereby higher levels of PA may ameliorate the effects of sedentary behavior on obesity [17]. Whereas, other studies have not found this interaction [18, 19]. Clarifying the relationship between sedentary behavior, PA and obesity is important for prioritizing prevention strategies. For example, if sedentary behavior is an independent determinant of obesity, then reducing sedentary time may be a more attainable health behavior goal in the long-term than increasing time spent in moderate-vigorous PA [20]. Given the sex differences in both PA (i.e., men have a higher odds of meeting PA recommendations), and sedentary behavior (i.e., men tend to accrue more sedentary time per day), it is plausible that the interactive relationship between PA and sedentary behavior on obesity markers may vary by sex [21, 22].

To address these knowledge gaps regarding sedentary behavior’s role in the obesity epidemic and how PA and sex may alter this role, we investigated the association between sedentary time expressed as hours of TV viewed per week and percent body fat (%BF) and the influence of PA and sex (meeting or not meeting PA guidelines) on this association in a diverse sample of adults. Clarity in this area will inform the extent to which sedentary behavior may be a viable target for obesity prevention efforts.

## Methods

### Study design

Data used for this study were collected during the Kansas City Built Environment and Health Study (KC BEST) [23, 24]. Briefly, KC BEST used a three-group nested (within U.S. Census block groups), cross-sectional design and a sampling scheme to ensure maximum income variations, independence of the environmental data, and adequate ethnic representation. Face-to-face, 60-min interviews were conducted by trained personnel at a minimum of 25 households in each of the 21 U.S. Census block groups included in the study. Individuals were eligible to participate if they met the following criteria: 1) between 18 and 74 years of age; 2) lived in the area at least 12 months; 3) able to read and understand surveys in English; and 4) primarily responsible for making food decisions for the household. Pregnant women and individuals who currently had any chronic health conditions or disabilities that prevented them from participating in PA were excluded from participation. One eligible individual per household was interviewed, and the justification behind interviewing the person who was primarily responsible for making household food decisions was that the main study, KC BEST, focused on food preparation and selection. Consent was obtained from all participants. Procedures were approved by the University of Missouri-Kansas City’s Institutional Review Board for the protection of human subjects and were performed in accordance with the ethical standards as per the 1964 Declaration of Helsinki and its later amendments or comparable ethical standards.

A total of 568 participants completed a face-to-face interview and, of these, 454 (80%) had complete data for all variables examined in this study and were included in the analyses. No significant differences in study variables were noted between included and excluded participants (*t* value ranged: *t* = 0.08; *p* = 0.94 for perceived stress to *t* = 1.9; *p* = 0.06 for TV h/wk. and Chi Square value ranged: *χ*^2^ = 0.01; p = 0.94 for PA category to *χ*^2^ = 3.17; *p* = 0.10 for education level).

### Measures

#### Percent body fat

Foot-to-foot bioelectrical impedance analysis (BIA) (Tanita TBF-300, Tokyo, Japan) was used to assess %BF during the face-to-face interviews. Participants were measured wearing light clothing and were instructed to stand barefoot with heel and forefoot placed on the metal electrode plates of the analyzer. All measurements were completed by a trained investigator according to the device manufacturer’s instructions. The Tanita 300 demonstrated strong evidence of concurrent validity (*r* = 0.94; *P <* 0.001) when compared with the “criterion standard” of dual energy x-ray absorptiometry (DEXA) for %BF [25].

#### Physical activity

The International PA Questionnaire (IPAQ) was used to quantify the time participants spent walking, and doing moderate and vigorous PA within the past 7 days. Participants were categorized as meeting PA guidelines if they reported engaging in at least one of the following: (1) three or more days of vigorous intensity PA [Metabolic Equivalent (MET) ≥ 8] of at least 25 min/d, (2) five or more days of moderate intensity PA (4–7 METs), (3) walking (3.3 METs) of at least 30 min/d each day, (4) five or more days of any combination of walking, moderate or vigorous intensity PA attaining at least 600 MET-min/wk. Participants not meeting PA guidelines were those who failed to meet any of the preceding criteria [26]. The IPAQ has been found to have adequate test-retest reliability (ρ = 0.81, 95% CI 0.79–0.82) and acceptable criterion validity (ρ = 0.33, 95% CI 0.26–0.39) when tested against accelerometers [27].

#### Sedentary behavior

The Sedentary Behavior Questionnaire (SBQ) for adults was used to assess time spent watching TV during the past week. Participants reported how much time they typically watched TV on a weekday and weekend day during the last 7 days. To arrive at TV h/wk., the weekday amount was multiplied by five and then added to the weekend amount which was multiplied by two. The SBQ has acceptable test-retest reliability [intraclass correlation coefficients between 0.828 and 0.857 for TV h/wk] and criterion validity (TV h/wk. with BMI: partial r = 0.16; *p* < 0.05) [28].

#### Sociodemographic characteristics

Self-reported sociodemographic characteristics included sex (female =0; male = 1), age in years, race/ethnicity category (non-minority = 0; minority = 1; minority included African Americans, Hispanics, Asians, native Hawaiian or other Pacific islander, or American Indian, Alaskan Native), marital status (married = 0; not married = 1), education level [high school (HS) diploma or less = 0; greater than a HS diploma = 1], employment status (unemployed = 0; employed = 1), and yearly median income [(low-income <$30,000/year = 0; middle-income $30,001 to $100,000/year = 1; high-income >$100,000/year = 2].

#### Depression symptoms

Symptoms of depression were measured using the 8-item Center for Epidemiologic Studies Depression (CES-D 8) scale. The response values were 4-point Likert scales, with a scoring range of 0 to 3 for each item, giving a total possible score range of 0 to 24. Higher scores indicated a higher frequency of depression symptoms. The CES-D 8 has comparable reliability estimates to those reported for the original version of the CES-D (Chronbach’s α = 0.92; r = 0.83) [29].

#### Perceived stress

The 4-item Perceived Stress Scale (PSS) was used to assess feelings and thoughts related to stress during the last month. Participants were asked to respond to each question using Likert scales that ranged from 0 to 4 giving a total possible score range from 0 to 16, with higher scores associated with greater perceived stress. The PSS has been found to be highly reliable in the general U.S. population [30].

#### Fruit and vegetable intake

The Block Fruit/Vegetable Screener was used to estimate weekly fruit and vegetable servings. Responses were categorized as: < 3 servings/wk. = 0; 4–6 servings/wk. = 1; ≥ 7 servings/wk. = 2. These self-report screeners have been highly correlated with actual intake (Spearman r values range from 0.6–0.7, *p* < .0001) [31].

#### Environment score

The PA Neighborhood Environment Survey (PANES) was used to assess perceptions about six aspects of the built environment thought to influence PA. A 4-point Likert scale with responses ranging from strongly disagree to strongly agree, was used for questions about the presence of transit stops, sidewalks, bicycling facilities, recreation facilities, and stores within walking distance. For the question on the main type of housing, response items were graded from low-density housing (single-family homes) to high-density housing (apartments or condos > 12 stories). For data analyses, responses were divided into two groups: disagree (strongly disagree and somewhat disagree = 0) and agree (strongly agree and somewhat agree = 1). For types of housing, single-family was coded 0 and all others coded 1. Thus, summary environment scores ranged from 0 to 6, with higher scores indicating a built environment more conducive for PA. Test–retest reliability for the PANES has been shown to range from *r* = 0.64 for free or low-cost recreation facilities to *r* = 0.84 for sidewalks on most streets [32].

### Analysis

Descriptive statistics were generated for all study variables and distributions checked for normality and corrected if necessary. Other assumptions (linearity, homoscedasticity, homogeneity of variance, multi-collinearity, and the presence of outliers) also were investigated and found to be within acceptable limits for the statistical tests used. The study was powered as a clustered, epidemiological survey. Thus, the number of clusters (U.S. Census block groups) was the primary driver for power. Power estimates indicated that a sample of 21 U.S. Census block-groups was needed to provide > 80% power to evaluate group differences in dichotomous outcomes and even greater power for continuous associations. Differences between groups (included vs. excluded participants and inactive vs. active) were examined using independent t-tests for continuous variables and Chi Square for categorical variables. Associations between the independent variables (sedentary behavior, PA, sex) and covariates (age, race/ethnicity, marital status, education, employment, yearly median income, environment score, depression symptoms, perceived stress, and fruit/vegetable intake) with %BF were examined using Pearson Product Moment correlation for continuous independent variables, Pearson point biserial correlation for dichotomous independent variables and one-way analysis of variance for multicategorical independent variables. Mixed models were generated to test the relationship between TV h/wk. and %BF and the extent to which this relationship was modified by PA level and sex while accounting for the multi-level nature of the data and covariates which were selected on the basis of being significantly (*P* < 0.05) correlated with %BF. Block group was designated as the random effect (with random intercept included in the models) and TV h/wk. and covariates were considered fixed effects in the models predicting %BF in the overall sample, by PA level, and by PA level within sex. The significance level was set at α < 0.05 and all analyses were conducted using the SPSS statistical software package (IBM Corp. Released 2015. IBM SPSS Statistics for Windows, Version 23.0. Armonk, NY: IBM Corp.)

## Results

### Participant characteristics

Of the 454 study participants, 70.9% were female, 26.4% reported a yearly household income of $30,000 or less, 33.9% were minority, 45.4% were not married, 23.8% had a HS education or less, and 36.8% were unemployed. Mean %BF for the sample was 33.2 ± 11.1 and 63.2% were classified as not meeting PA guidelines. Mean TV viewing was 19.3 ± 15.5 h/wk. Descriptive statistics for the full sample and stratified by sex and activity level can be found in Table 1.

**Table 1:** Sample characteristics, overall, and stratified by activity level and sex

	Overall Sample *n* = 454	Activity Level		Sex	
		Meeting PA guidelines *n* = 168	Not meeting PA guidelines *n* = 286	Female *n* = 322	Male *n* = 132
% Body fat [M (SD)]	33.2 (11.1)	31.0 (9.5)	34.5 (11.8)	36.0 (10.4)	26.4 (9.7)
TV viewing (h/wk) [M (SD)]	19.3 (15.5)	17.7 (13.6)	20.3 (16.5)	18.6 (15.8)	21.2 (14.8)
Age (y) [M (SD)]	44.4 (14.0)	43.3 (14.0)	45.0 (13.9)	44.2 (13.6)	44.8 (14.9)
Depression symptoms [M (SD)] (Possible range 0–24)	5.9 (5.9)	5.0 (5.5)	6.5 (6.0)	6.3 (6.0)	5.1 (5.4)
Perceived stress [M (SD)] (Possible range 0–16)	4.0 (3.0)	3.7 (2.8)	4.1 (3.1)	3.9 (3.1)	4.0 (2.7)
Environment score [M (SD)] (Possible range 0–6)	3.3 (1.4)	3.4 (1.5)	3.2 (1.4)	3.2 (1.4)	3.4 (1.5)
Female (%)	70.9	68.5	72.4	–	–
Race/ethnicity (%)					
Non-Minority	66.1	27.4	37.8	63.7	72.0
Minority	33.9	72.6	62.2	36.3	28.0
Income (%)					
< $30,000/year	26.4	17.3	31.8	26.7	25.8
$30,001 to $100,000/year	58.0	56.5	58.7	59.6	53.8
> $100,000/year	15.6	26.2	9.4	13.7	20.5
Educational attainment (%)					
High school or less	23.8	19.0	26.6	24.8	21.2
Greater than high school	76.2	81.0	73.4	75.2	78.8
Marital status (%)					
Married	54.6	58.3	52.4	56.2	50.8
Not married	45.4	41.7	47.6	43.8	49.2
Employment status (%)					
Employed	63.2	35.1	37.8	61.2	68.2
Not employed	36.8	64.9	62.2	38.8	31.8
Activity level (%)					
Not meeting PA guidelines	63.2	–	–	64.6	59.8
Meeting PA guidelines	36.8	–	–	35.4	40.2
Fruit and Vegetable Intake (%)					
< 3 servings/week	7.7	4.2	9.8	8.1	6.8
4 to 6 servings/week	50.1	44.3	53.5	44.9	62.9
7 or more servings/week	42.2	51.5	36.7	47.0	30.3

### Relationships between study variables and %BF

Hours of TV viewed per week (r = .17) were positively correlated with %BF, while meeting PA guidelines (r = −.17), and male (r = −.40) were negatively related to %BF (all *P* values <.01). Among the study covariates, higher levels of education (r = −.12), and living in an environment more conducive to PA (r = −.15) were significantly related to lower %BF. Being a minority (r = .16), older (r = .15), and reporting higher depressive symptoms (Pearson r = .15), and greater perceived stress (r = .10), were also correlated with having a higher %BF (all P values <.05; see Table 2 for a full listing). Percent body fat did not differ across yearly median income categories [F(2,453) = 2.62;*p* = .07] or fruit/vegetable intake categories [F(2,452) = 1.12;*p* = .34].

**Table 2:** Zero-order correlations between study variables and %BF

Study Variables	Pearson’s r
TV h/wk	.17***
PA category (not meeting PA guidelines = 0; meeting PA guidelines = 1)	−.15**
Sex (female = 0; male =1)	−.40***
Age y	.15**
Race/ethnicity (non-minority = 0; minority = 1)	.16**
Marital status (married =0; not married =1)	.04
Education (<HS = 0; >HS =1)	−.12*
Employment (unemployed = 0; employed =1)	−.05
Environment score	−.15**
Depression symptoms	.15**
Perceived stress	.10*

*p < 0.05;**p < 0.005;***p < 0.001

### Multivariate associations between TV viewing and %BF

In a mixed-model generated to assess the independent association between TV h/wk. and %BF using the full sample, TV h/wk. were positively and significantly associated with %BF (β = 0.86; SE = .28; *p* < 0.05; Table 3).

**Table 3:** Mixed model for the overall sample predicting %BF

	Coefficient	Standard error
Fixed effects		
Grand intercept	28.16***	2.54
TV (h/wk)	.85**	.28
Age (y)	.12***	.03
Race/ethnicity	2.19*	1.03
Education level	−1.23	1.13
Environmental score	−.49	.33
Depression symptoms	.13	.10
Perceived stress	.17	.20
Physical Activity Level	− 1.90*	.96
Sex	−9.40***	1.02
Random effects		
Level 1 variance	92.62***	6.43
Level 2 variance	.98	2.22
Model fit		
AIC	3345	
*n* (individuals)	454	
*k* (block groups)	21	

*p < 0.05;**p < 0.005;***p < 0.001. AIC – Akaike’s Information Criterion; Level 1 is the residual variance and Level 2 is the variance for intercept across block group

To test the modifying effects of PA, the overall mixed model was stratified by PA level (Table 4). Results showed that among participants not meeting PA guidelines, TV h/wk. were significantly associated with %BF such that for each additional h of TV watched per wk., a significant increase in %BF of 1.03% (e.g., going from a %BF of 20.0 to 21.03%) was observed after holding other variables in the model constant (β =1.03; SE = 0.37, *p* < .005). Among participants meeting PA guidelins, the relationship between TV h/wk. and %BF remained non-significant (β = .16; SE = .44; *p* = 0.73). Several covariates were also significantly associated with %BF. Specifically, age (β = .17; SE = .05; *p* < .001), and sex (β = − 9.91; SE = 1.39; *p* < .001) were associated with %BF in participants not meeting PA guidelines while only sex was significantly associated with %BF in participants meeting PA guidelines (β = − 7.88; SE = 1.53; *p* < 0.001).

**Table 4:** Mixed models predicting %BF in inactive and active subjects

	Not meeting PA guidelines		Meeting PA guidelines	
	Coefficient	Standard error	Coefficient	Standard error
Fixed effects				
Grand intercept	25.01****	3.33	33.56****	3.80
TV (h/wk)	1.03***	.37	.15	.44
Age (y)	.17****	.05	.06	.05
Race/ethnicity	2.13	1.35	2.46	1.55
Education level	−.56	1.46	−3.40	1.86
Environmental score	−.58	.45	−.55	.48
Depression symptoms	.16	.14	.07	.16
Perceived stress	.22	.27	.17	.32
Sex	−9.91****	1.39	−7.96****	1.53
Random effects				
Level 1 variance	105.05****	9.20	71.11****	8.66
Level 2 variance	3.64	2.78	2.01	4.43
Model fit				
AIC	2147		1182	
*n* (individuals)	287		167	
*k* (block groups)	21		21	

*p < 0.05;**p < 0.01;***p < 0.005;****p < 0.001. AIC – Akaike’s Information Criterion; Level 1 is the residual variance and Level 2 is the variance for intercept across block group

**Fig. 1:**
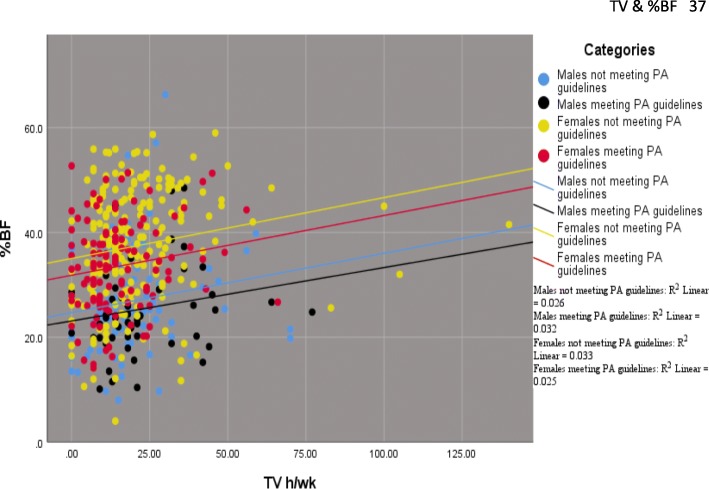
Relationship between TV h/wk. and %BF in male and female subjects who met and didn’t meet PA guidelines

In order to examine sex differences in the relationship between TV viewing, PA, and %BF, the mixed models were further stratified by sex (Table 5). These data indicated that among females not meeting PA guidelines, for every additional h of TV viewed per wk., there was a corresponding increase in %BF of 1.14% (β = 1.14; SE = .43; *p* < .01). No association between TV h/wk. and %BF was seen for females or males meeting PA guidelines. Of note is that the random effects for block group (level 2 variances) were negligible in all models indicating that only a small portion of the variance in %BF was accounted for by latent factors associated with block groups. Figure 1 provides a depiction of the relationship between TV, %BF, sex and PA level. As can be seen, inactive females had higher %BF than active females at any given dose of TV.

**Table 5:** Mixed models predicting %BF in females and males by PA category

	Female				Male			
	Not meeting PA guidelines		Meeting PA guidelines		Not meeting PA guidelines		Meeting PA guidelines	
	Coef.	SE	Coef.	SE	Coef.	SE	Coef.	SE
Fixed effects								
Grand intercept	22.38****	3.91	29.89****	5.58	20.34***	6.00	30.67****	6.99
TV (h/wk)	1.14**	.43	−.18	.53	.34	.71	.95	.81
Age (y)	.20****	.06	.09	.06	.12	.08	−.03	.09
Race/ethnicity	4.06**	1.51	2.42	1.94	−6.54*	2.78	1.22	2.59
Education level	.56	1.73	−3.43	2.15	−2.48	2.61	−3.18	3.47
Environmental score	−.83	.54	.04	.58	−.01	.76	−.73	.87
Depression symptoms	.20	.16	−.10	.17	−.26	.27	.85*	.35
Perceived stress	.12	.30	.81*	.34	1.12	.58	−1.72**	.63
Random effects								
Level 1 variance	103.89****	10.39	66.45****	10.53	99.73****	16.73	60.51****	15.76
Level 2 variance	3.51	8.01	6.54	8.55	3.58	16.91	3.33	11.48
Model fit								
AIC	1552		803		575.8		357.2	
*n* (individuals)	208		114		79		53	
*k* (block groups)	21		21		21		21	

*p < 0.05;**p < 0.01;***p < 0.005;****p < 0.001. AIC – Akaike’s Information Criterion; Coef

Coefficient; SE – Standard Error. Level 1 is the residual variance and Level 2 is the variance for intercept across block group

## Discussion

The purpose of this study was to investigate the association between TV viewing time and %BF, and the extent to which this association varied as a function of PA level and sex. The main findings were that meeting PA guidelines ameliorated the significant, positive relationship between increased TV viewing and %BF. Moreover, among females not meeting PA guidelines, every additional h of TV viewed per wk. was independently and significantly associated with a 1.14% increase in %BF. These data substantially contribute to a complex literature reporting on the interplay between sedentary behavior, PA and sex by suggesting that reduced TV viewing time and/or increased PA may be particularly important for lowering cardiometabolic disease risk in females.

Our data showed that for each additional h of TV viewed per wk., adults not meeting PA guidelines displayed an increase in %BF of 1.06%; by contrast, no such association was seen in adults meeting PA guidelines. The implication that higher levels of PA may ameliorate the positive association between TV viewing and %BF adds to a mixed body of evidence. Some studies have shown higher levels of TV viewing to be significantly associated with overweight status independent of PA levels and other confounders such as sex and age [16, 33–35]. For example, Menai and colleagues [35] reported that in 2517 adults who completed two assessments six-years apart (2001 and 2007), a one h/d increase in TV viewing was associated with a significant, 0.28% increase in body fat mass, irrespective of PA and demographic factors. By contrast, other studies show no significant relationship between TV viewing and markers of overweight/obesity once PA levels are considered in the multivariable models [12, 36, 37]. Stratification by meeting versus not meeting PA levels allowed us to add some clarity to this mixed body of evidence by suggesting that higher levels of TV time impact %BF fat only in adults not meeting PA guidelines, thus affording a more nuanced understanding of these complex relationships.

Upon further stratification by sex, our results showed that the positive association between h of TV viewed per wk. and %BF was only significant for females not meeting PA guidelines. Sex differences in the association between sedentary time and body weight markers have been previously shown [38, 39]. For instance, overall sedentary time has been positively associated with body mass index (BMI) in females but not males, whereas, longer sitting time at work has been significantly associated with higher BMI in men, but not women [40–42]. This lack of concordance in the literature regarding sex differences in the association between sedentary time and %BF markers may be partially attributed to the complex nature of sedentary behaviors. Although sedentary behavior is defined as any waking behavior characterized by an energy expenditure ≤1.5 metabolic equivalents (METs), while in a sitting, reclining or lying posture, this biological operationalization belies a range of behavioral activities (i.e., reading, computer use), and contexts (i.e., commuting, workplace, home), that can in turn alter the duration of sedentary behavior bouts (i.e., period of uninterrupted sedentary time) and sedentary time interruptions (i.e., a non-sedentary bout in between two sedentary bouts) [6]. Sex differences in these behavioral and contextual sedentary time variables are not yet understood. Given that shorter sedentary behavior bouts and greater interruptions have been associated with reduced cardiovascular disease risk, it could be that sex differences in TV viewing behaviors contribute to the significant association between TV viewing and %BF among inactive females [43]. Relevant to this line of discussion is the moderating role of eating behaviors in the relationship between TV viewing and %BF. Data show that frequent consumption of calorie-dense snack and fried foods while watching TV accentuates the association between TV viewing and increased body fatness [44, 45]. It could be that the females in our sample were more likely to eat calorie-dense foods than the males. Future studies are needed to elucidate gender differences in snacking while watching TV.

One of the key clinical and population health implications from this study is that reducing TV viewing time should be more widely regarded as a cardiometabolic risk behavior, particularly for women not meeting PA guidelines. That the average adult watches almost five h/d of TV and that TV viewing is associated with greater food intake, poorer dietary intake, and poorer sleep health, underscores this premise. While the efficacies of several interventions to reduce TV viewing and screen-time to lower BMI and curb weight gain in pediatric and adolescent populations have been examined, considerably fewer such studies have been carried out in adult populations [46–54]. Otten and colleagues [54] found that 20 overweight adults using an electronic lock-out system for 6 weeks displayed greater decreases in BMI than a group of observation only controls. However, in a larger study on adults (*N* = 153) where households were randomized to a home-based obesity intervention that involved TV-limiting devices, less TV viewing was not associated with a significant decrease in BMI 1 year later [55]. The current study adds to the literature in this area and suggests that effective clinical and population-level strategies are necessary to address TV viewing, an important health risk behavior.

Findings from this study should be interpreted with consideration of some design, measurement, and data limitations including the fact that the study was cross-sectional and so precludes the consideration of the temporal relationship between the study variables. In terms of measurement, TV viewing was the only sedentary behavior assessed and it, along with other key study variables (i.e., PA) were not objectively measured. Moreover, sleep health and tobacco use are key variables shown to relate to PA and body composition, but they were not considered in the current study [56–58]. The use of BIA for assessing %BF typically requires adherence to set guidelines particularly concerning body water content (hydration status) [59]. However, given the large scale nature of this study with respect to directly measuring participants weight, height, %BF, conducting nearly 1 hour interviews about their health behaviors, and then also directly measuring the surrounding built environment, it was not logistically possible or economically feasible to standardize the times the interviews were conducted or to ensure proper hydration status at the time of the interview. We believe that BIA was the preferred choice (based on practicality and performance) for assessing %BF in a large-scale, epidemiological study such as this one. From a data perspective, the reported relationships yielded small correlations and explained a relatively small percentage of the variance in %BF; however, this is common in studies examining TV viewing and weight indicators [60–62].

Despite these limitations, the current study has strengths worth noting. First, a multi-level, analytical approach was used to account for any effects representing unobserved (i.e., latent) block group-level characteristics that could have affected individual-level outcomes. Second, aside from the limitation mentioned above, objective assessments of body composition were obtained using state-of-the art, high-grade equipment that provides measures of %BF comparable to those obtained with DEXA [25]. Lastly, the data were collected during in-person interviews conducted at each participant’s place of residence. No other studies on sedentary behavior and body composition have used this methodology. Besides having several advantages over phone interviews (e.g., more representative of residents in low-income areas, allow for use of visuals, verification of certain demographics, and the elimination of “dead-end” selections, e.g., non-working telephone numbers), door-to-door interviews can reach a pool of study participants that may not be captured by traditional data collection techniques requiring study participants to travel to a data collection location (e.g., lab, community center) [63–65]. These individuals may express unique characteristics relevant to examining sedentary behaviors. For example, in a previous study conducted in similar block groups in the same city as the current study, we found that respondents to door-to-door surveys reported sitting an average of 331 min/wk. while respondents to the same survey administered at centralized health fairs held in the same block groups reported sitting an average of 217 min/wk. (*p* < .01) [66]. Therefore, the current study may provide a missing piece of the spectrum of sedentary behavior (i.e., reduced truncation of the sedentary time distribution), thus improving analytics and providing a more accurate picture of the association between sedentary behavior and a health status indicator. This is similar to LaPorte and colleagues’ contention (1984) regarding the relationship between PA and cardiovascular disease [67].

## Conclusions

Our findings indicated that time spent watching TV and engaging in PA are both important from a cardio-metabolic disease prevention perspective. Future studies to verify these associations prospectively utilizing objective assessment of multiple sedentary behaviors and PA domains across different contexts are warranted. The development and testing of accessible and effective strategies to increase PA and reduce TV viewing should be embraced as an approach to reducing excess BF accumulation especially among inactive women.

## Data Availability

The datasets used and/or analyzed during the current study are available from the corresponding author on reasonable request.

## Abbreviations

%BF: percent body fat
AIC: Akaike’s Information Criterion
BMI: body mass index
CES-D 9: Center for Epidemiologic Studies Depression
Coef.: Coefficient.
DEXA: dual energy x-ray absorptiometry
h: hours
HS: high school
IPAQ: International Physical Activity Questionnaire
KC BEST: Kansas City Built Environment and Health Study
min: minutes
PA: physical activity
PANES: Physical Activity Neighborhood Environment Survey
PSS: Perceived Stress Scale
SD: standard deviation
SE: standard error
SPSS: Statistical Package for the Social Sciences
TV: television
U.S.: United States
wk: week
